# SIDT1-dependent absorption in the stomach mediates host uptake of dietary and orally administered microRNAs

**DOI:** 10.1038/s41422-020-0389-3

**Published:** 2020-08-17

**Authors:** Qun Chen, Fan Zhang, Lei Dong, Huimin Wu, Jie Xu, Hanqin Li, Jin Wang, Zhen Zhou, Chunyan Liu, Yanbo Wang, Yuyan Liu, Liangsheng Lu, Chen Wang, Minghui Liu, Xi Chen, Cheng Wang, Chunni Zhang, Dangsheng Li, Ke Zen, Fangyu Wang, Qipeng Zhang, Chen-Yu Zhang

**Affiliations:** 1 Nanjing Drum Tower Hospital Center of Molecular Diagnostic and Therapy, State Key Laboratory of Pharmaceutical Biotechnology, Jiangsu Engineering Research Center for MicroRNA Biology and Biotechnology, NJU Advanced Institute of Life Sciences (NAILS), NJU Institute of AI Biomedicine and Biotechnology, School of Life Sciences, Nanjing University, Nanjing, Jiangsu 210023 China; 2 Department of Gastroenterology and Hepatology, Jinling Hospital, Medical School of Nanjing University, Nanjing, Jiangsu 210002 China; 3 Department of Clinical Laboratory, Jinling Hospital, School of Medicine, Nanjing University, Nanjing, Jiangsu 210002 China; 4 Shanghai Institute of Biochemistry and Cell Biology, Center for Excellence in Molecular Cell Science, Chinese Academy of Sciences, Shanghai, 200031 China

**Keywords:** miRNAs, RNA transport

## Abstract

Dietary microRNAs have been shown to be absorbed by mammals and regulate host gene expression, but the absorption mechanism remains unknown. Here, we show that SIDT1 expressed on gastric pit cells in the stomach is required for the absorption of dietary microRNAs. SIDT1-deficient mice show reduced basal levels and impaired dynamic absorption of dietary microRNAs. Notably, we identified the stomach as the primary site for dietary microRNA absorption, which is dramatically attenuated in the stomachs of SIDT1-deficient mice. Mechanistic analyses revealed that the uptake of exogenous microRNAs by gastric pit cells is SIDT1 and low-pH dependent. Furthermore, oral administration of plant-derived miR2911 retards liver fibrosis, and this protective effect was abolished in SIDT1-deficient mice. Our findings reveal a major mechanism underlying the absorption of dietary microRNAs, uncover an unexpected role of the stomach and shed light on developing small RNA therapeutics by oral delivery.

## Introduction

Recent studies have shown that intact plant microRNA (miRNA) in foods can be absorbed through the mammalian digestive system and mediate cross-kingdom gene regulation.^1–5^ These reports provide new insight into the oral administration of RNA therapeutic drugs, as the oral delivery of naked RNA is thought to be an invincible challenge in RNA drug development because large hydrophilic molecules, such as RNAs, are believed to be completely blocked from penetrating cells by the lipid bilayer of the plasma membrane.^6,7^ Additionally, according to the classic digestion physiology, nucleic acids are hydrolyzed to oligonucleotides, nucleotides, nucleosides, and even free bases in the small intestine by enzymes (nucleases, nucleotidases and nucleosidases) and then absorbed as materials that lack sequence-based biological function.^8–10^ Therefore, the mechanism by which intact dietary miRNAs travel across the mammalian gastrointestinal (GI) tract, thus being functionally transferred from plants to mammals, is still unknown.

A plasma membrane protein, systemic RNA interference defective protein 1 (SID-1), is responsible for the transport of exogenous double-stranded RNA (dsRNA) into the cytoplasm and is therefore essential for systemic RNA interference in *Caenorhabditis elegans*.^11–15^ SID-1, which harbors a domain containing eleven predicted transmembrane helices and acts in a multimeric form, transports dsRNA of different lengths^13,14^ and partially double-stranded RNA molecules, such as miRNA precursors and hairpin RNAs.^15^ Mammalian SID-1 transmembrane family member 1 (SIDT1), as its nematode homolog SID-1, also localizes to the plasma membrane^16^ and mediates intercellular miRNA transport^17^ as well as extracellular small interfering RNA (siRNA) uptake.^16^ Based on the above findings, we hypothesized that SIDT1 mediates the absorption of dietary miRNAs in mammals.

In the present study, we tested how and where SIDT1 performs its functions. We identified SIDT1 as an RNA transporter that mediates dietary miRNA absorption in the mammalian stomach. The stomach’s highly acidic environment is crucial for SIDT1-dependent absorption of miRNAs. Notably, dietary miRNAs absorbed via SIDT1 can exert biological functions in the host, and oral administration of plant-derived miR2911 retards liver fibrosis, which is abolished by SIDT1 deficiency.

## Results

### Dynamic absorption of synthetic miRNA is compromised in *Sidt1*-knockout mice

To assess whether SIDT1 mediates the absorption of dietary miRNAs, we employed a SIDT1-deficient (*Sidt1*^−/−^) mouse in this study. The *Sidt1*^−/−^ mouse was generated by replacing the first three exons of the *Sidt1* coding region with a lacZ reporter (Supplementary information, Fig. S1a), and the loss of *Sidt1* expression was validated in various organs by using reverse transcription PCR (Supplementary information, Fig. S1b).

**Fig. 1:**
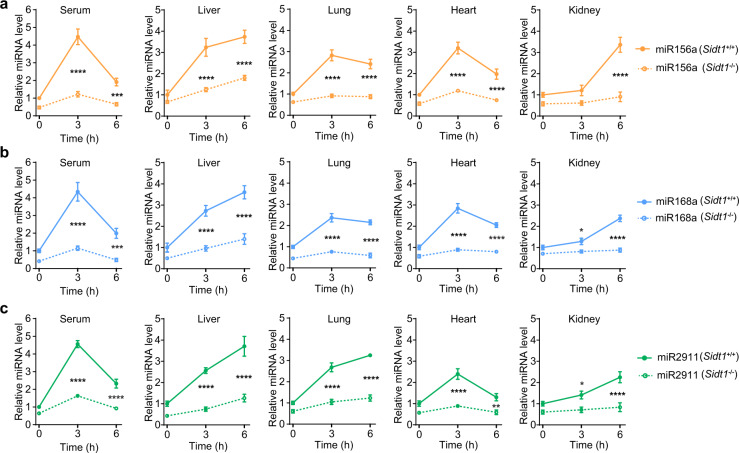
Dynamic absorption of dietary miRNAs in *Sidt1*^+/+^ or *Sidt1*^−/−^ mice. **a**–**c** Levels of miR156a (**a**), miR168a (**b**) or miR2911 (**c**) in serum (*n* = 10) and internal organs (*n* = 6) of *Sidt1*^*+/+*^ or *Sidt1*^−/−^ mice at the indicated time points after gavage feeding of synthetic dietary miRNAs. Two-way ANOVA with Sidak’s post hoc test; *P < 0.05, **P < 0.01, ***P < 0.001, ****P < 0.0001.

To test whether loss of *Sidt1* could impair dietary miRNA absorption, three representative miRNAs (miR156a,^5^ miR168a,^1^ and miR2911^2^) were synthesized and fed by gavage to the *Sidt1*^+/+^ and *Sidt1*^−/−^ mice. As shown in Fig. 1, compared to *Sidt1*^+/+^ mice, markedly diminished miRNA absorption was observed in the serum, liver, lung, heart, and kidney from *Sidt1*^−/−^ mice after gavage feeding of synthetic dietary miRNAs. Furthermore, reduced absorption of exogenous animal miRNA, *Lottia gigantea* lgi-miR-133-5p, was also observed in *Sidt1*^−/−^ mice (Supplementary information, Fig. S1c).

### SIDT1 mediates the absorption of physiological miRNAs derived from natural foods

**Fig. 2:**
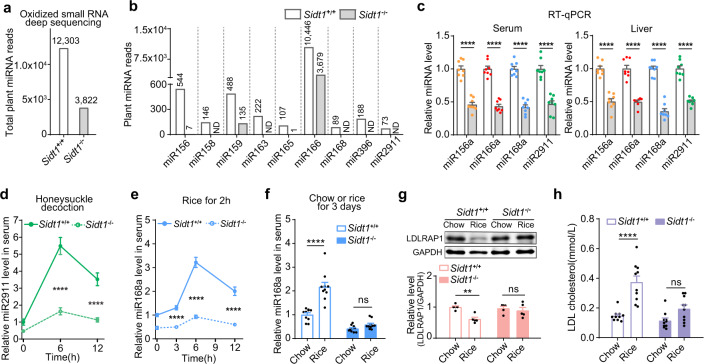
SIDT1 mediates the absorption of physiological miRNAs derived from natural foods. **a**, **b** Number of total reads (**a**) and reads of individual oxidized plant miRNAs (**b**) in livers of *Sidt1*^*+/+*^ or *Sidt1*^−/−^ mice detected by small RNA deep sequencing. ND, not detectable. **c** Levels of individual dietary plant miRNAs in the serum and livers of mice (*n* = 8) measured by RT-qPCR. Two-tailed Student’s *t-*test. ****P < 0.0001. **d** Serum miR2911 levels in *Sidt1*^*+/+*^ or *Sidt1*^*–/–*^ mice (*n* = 6) at the indicated time points after gavage feeding of honeysuckle decoction. Two-way ANOVA with Sidak’s post hoc test. ****P < 0.0001. **e** Serum miR168a level in *Sidt1*^*+/+*^ or *Sidt1*^−/−^ mice (*n* = 9) at the indicated time points following rice feeding for 2 h. Two-way ANOVA with Sidak’s post hoc test. ****P < 0.0001. **f**–**h** Levels of serum miR168a (**f**, *n* = 10), liver LDLRAP1 (**g**, *n* = 5) and serum LDL cholesterol (**h**, *n* = 10) in *Sidt1*^*+/+*^ or *Sidt1*^−/−^ mice after chow or rice feeding for 3 days. Two-way ANOVA with Sidak’s post hoc test. ns, not significant, **P < 0.01, ****P < 0.0001.

To compare the basal levels of diet-derived plant miRNAs in *Sidt1*^+/+^ and *Sidt1*^−/−^ mice, we performed oxidized-RNA deep sequencing and found that the total number of plant miRNA reads (Fig. 2a) and the number of each detected individual plant miRNA reads (Fig. 2b) were all reduced in the liver of *Sidt1*^−/−^ mice. The reduction in miR156a, miR166a, miR168a, and miR2911 levels was further verified by quantitative real-time reverse transcription PCR (RT-qPCR) in the serum and liver of *Sidt1*^−/−^ mice (Fig. 2c). In contrast, loss of *Sidt1* did not affect endogenous mammalian miRNA levels in the serum or liver (Supplementary information, Fig. S1d). These results indicated that *Sidt1*^−/−^ mice had a reduced basal level of dietary plant miRNAs.

Interestingly, similar miRNA absorption kinetics were observed when the mice were fed miR2911-enriched honeysuckle decoction^2^ (Fig. 2d) or miR168a-enriched rice^1^ (Fig. 2e), showing dramatically lower serum levels of miR2911 (Fig. 2d) and miR168a (Fig. 2e) in *Sidt1*^−/−^ mice. A previous study showed that rice-derived miR168a decreases low-density lipoprotein receptor adapter protein 1 (LDLRAP1) levels in mouse liver, which leads to elevated plasma levels of LDL cholesterol.^1^ Consistent with this finding, compared with the mice fed the chow diet, we observed elevated serum miR168a levels, reduced hepatic LDLRAP1 protein expression, and increased serum LDL cholesterol levels in rice-fed *Sidt1*^+/+^ mice but not *Sidt1*^−/−^ mice (Fig. 2f–h). Body weight and food intake were comparable between *Sidt1*^+/+^ and *Sidt1*^−/−^ mice (Supplementary information, Fig. S1e, f), suggesting that the changes in serum miR168a levels were not due to changes in appetite. Taken together, these results indicate that lack of SIDT1 impairs dietary miRNA absorption.

### SIDT1 is expressed in pit cells of the stomach epithelium and localizes to the plasma membrane

**Fig. 3:**
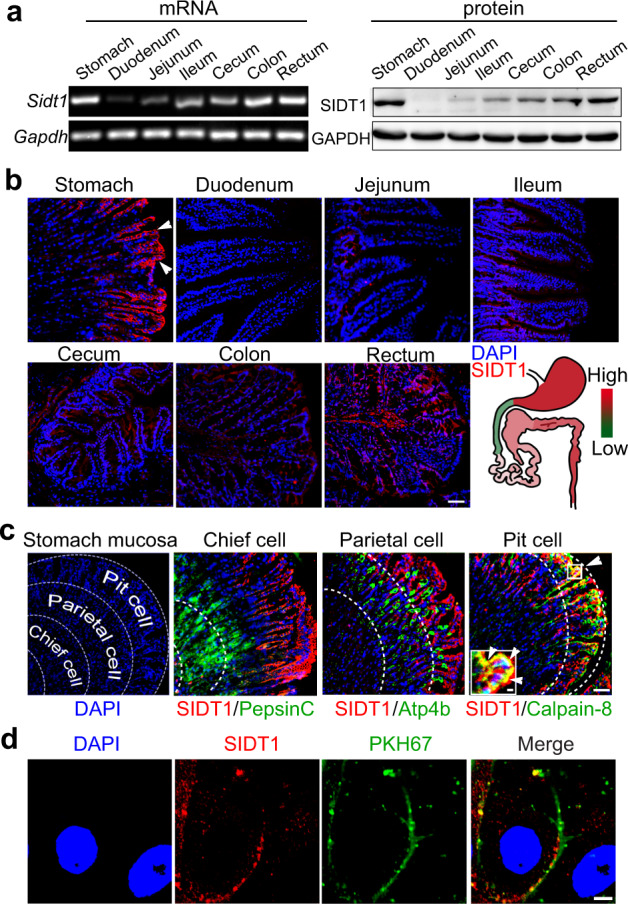
SIDT1 is expressed in pit cells of the stomach epithelium and localizes to the plasma membrane. **a** Expression of *Sidt1* mRNA (left) and SIDT1 protein (right) along the mouse GI tract. **b** Representative images and heatmap summary of SIDT1 immunostaining along the mouse GI tract. Scale bar, 50 μm. **c** Expression and localization of SIDT1 in the mouse gastric mucosa by costaining with gastric cell subtype markers. Scale bars, 50 μm and 5 μm (inset). **d** Subcellular localization of SIDT1 in mouse PGECs by costaining with PKH67, a cell membrane-labeling dye. Scale bar, 5 μm.

The GI tract is the organ system that controls food intake, digestion and absorption. To ascertain the organ(s) involved in SIDT1-mediated miRNA absorption, we dissected the GI tract and analyzed SIDT1 expression along the GI tract. Interestingly, SIDT1 mRNA and protein were mainly detected in the stomach and large intestine (including the cecum, colon and rectum) but not in the small intestine (especially the duodenum) (Fig. 3a, b; Supplementary information, Fig. S2). Notably, SIDT1 was mainly located in gastric surface mucous cells (pit cells) but barely expressed in the parietal or chief cells (Fig. 3c). Immunofluorescence staining of the mouse primary gastric epithelial cells (PGECs) showed colocalization of SIDT1 and the plasma membrane (Fig. 3d), suggesting that SIDT1 comes into direct contact with dietary miRNAs.

### SIDT1-mediated dietary miRNA absorption occurs in the stomach

**Fig. 4:**
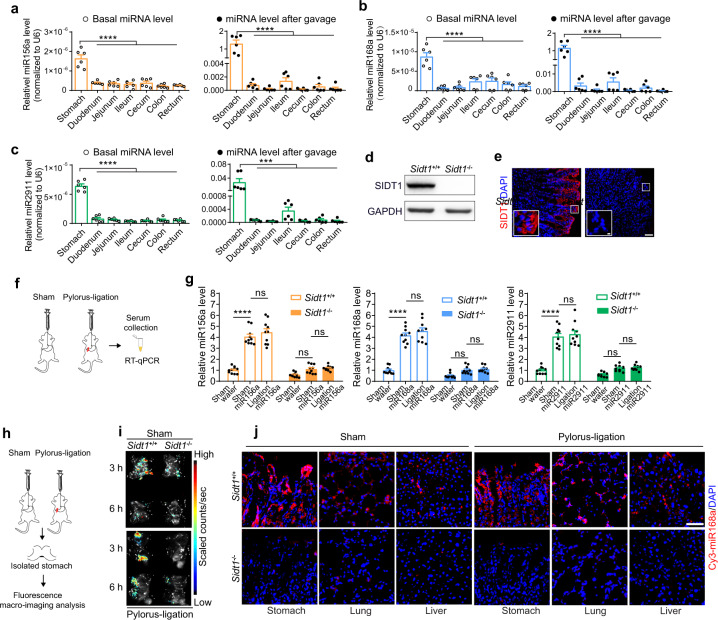
SIDT1-mediated dietary miRNA absorption occurs in the stomach. **a**–**c** Basal (left) and post-gavage feeding (right) levels of miR156a, miR168a and miR2911 along the mouse GI tract (*n* = 6). One-way ANOVA with Tukey’s post hoc test. ***P < 0.001, ****P < 0.0001. **d**, **e** SIDT1 levels in the stomachs of *Sidt1*^*+/+*^ or *Sidt1*^−/−^ mice detected by western blotting (**d**) and immunofluorescence staining (**e**). Scale bars, 50 μm and 5 μm (inset). **f** Diagram of the pylorus ligation experiment. **g** Serum levels of miR156a, miR168a and miR2911 in sham or pylorus-ligated mice at 3 h post gavage feeding of synthetic dietary miRNAs or vehicle (*n* = 10). Two-way ANOVA with Sidak’s post hoc test. ns, not significant, ****P < 0.0001. **h** Diagram of ex vivo macroimaging analysis of isolated mouse stomachs from the pylorus ligation experiment. **i** Macroimaging of the stomachs from sham or pylorus-ligated mice at 3 h and 6 h post gavage feeding of Cy5-labeled miR168a. **j** Confocal microscopy imaging of Cy3-labeled miR168a (red) and DAPI (blue) in tissue sections from sham or pylorus-ligated mice at 3 h post gavage feeding. Scale bar, 50 μm.

To dissect the organ(s) responsible for dietary miRNA absorption in the GI tract, we examined miRNA levels in different GI tract parts. Consistent with the expression pattern of the SIDT1 protein, plant miR156a, miR168a and miR2911 were highly enriched in the stomach compared to other organs in the GI tract of mice on a regular chow diet or after gavage feeding of synthetic dietary miRNAs (Fig. 4a–c).

We validated the loss of the SIDT1 protein in the stomach of *Sidt1*^−/−^ mice (Fig. 4d, e). Then, we performed pylorus ligation surgery to retain food in the stomach (Fig. 4f) to further test whether SIDT1 mediates the absorption of miRNAs in the stomach. Oral administration of glucose increased the blood glucose level in the control group (sham) but not in the pylorus-ligated mice (Supplementary information, Fig. S3), indicating successful ligation. After gavage feeding of synthetic dietary miRNAs, we collected serum from the sham and ligation groups and measured the levels of these dietary miRNAs. In both the sham and ligation groups, the serum levels of all three miRNAs in *Sidt1*^+/+^ mice were significantly elevated, and the increase was comparable between the two groups (Fig. 4g), indicating that the intestine and other lower parts of the GI tract do not contribute significantly to this miRNA absorption process and that the stomach is the primary organ for dietary miRNA absorption. In addition, no increase in miRNA levels was observed in the serum of *Sidt1*^−/−^ mice (Fig. 4g), suggesting that SIDT1 is required for miRNA absorption in the stomach. To visualize the absorption of miRNAs, both the sham and ligation groups were fed Cy5-labeled miR168a, and their stomach tissues were collected for imaging analysis 3 and 6 h after feeding (Fig. 4h). Cy5 fluorescent signals were detected in the stomach of *Sidt1*^+/+^ mice in both the sham and ligation groups (Fig. 4i). In contrast, the fluorescent signals in the stomach of *Sidt1*^−/−^ mice were dramatically lower than those in *Sidt1*^+/+^ mice (Fig. 4i). To further validate the dietary miRNA absorption and secretion via the circulation system, we collected freshly fixed tissues and examined the fluorescent signals after gavage feeding of Cy3-labeled miR168a. Interestingly, in addition to the stomach, fluorescent signals were observed in the liver and lungs of *Sidt1*^+/+^ mice in both the sham and ligation groups but not in the corresponding tissues of *Sidt1*^−/−^ mice (Fig. 4j), suggesting that miRNAs absorbed via SIDT1 in the stomach were transmitted to other tissues. Taken together, the above results indicate that the stomach is the primary organ that absorbs miRNAs and that gastric SIDT1 mediates this absorption.

### Exogenous miRNAs are taken up by PGECs through SIDT1 in a pH-dependent manner

**Fig. 5:**
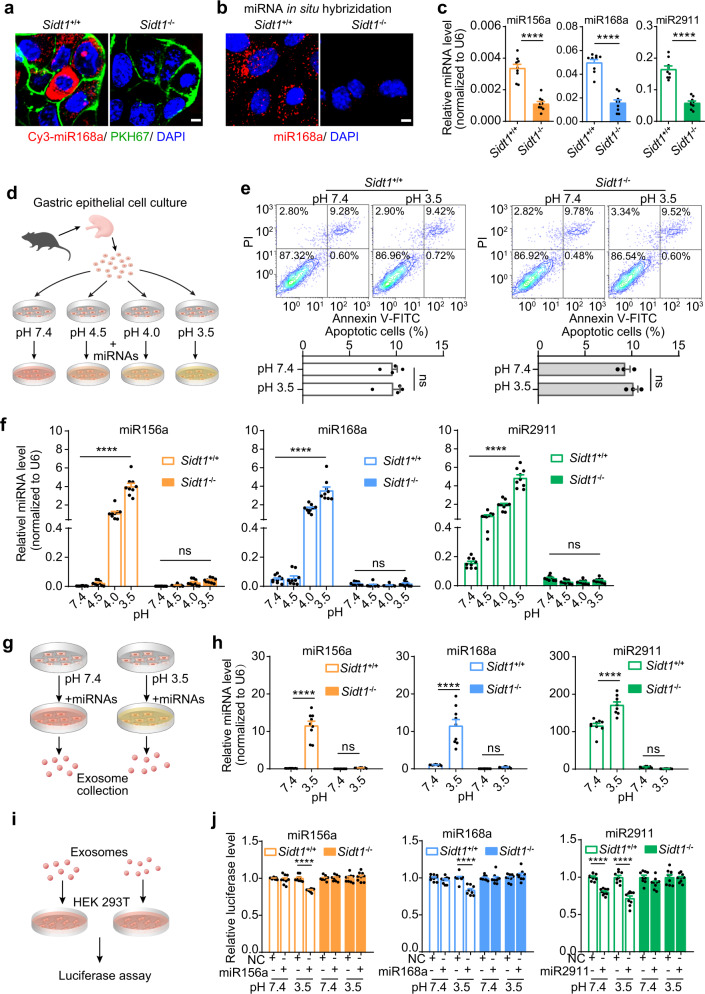
Exogenous miRNAs are taken up by PGECs through SIDT1 in a pH-dependent manner. **a**–**c** Uptake of synthetic miRNAs by *Sidt1*^+/+^ and *Sidt1*^−/−^ mouse PGECs after 30-min incubation with synthetic miRNAs at pH 7.4. Cy3-conjugated miR168a detected by direct fluorescence microscopy (**a**; scale bar, 5 μm), miR168a by in situ hybridization (**b**; scale bar, 5 μm), and miR168a, miR156a and miR2911 by RT-qPCR (**c**, *n* = 9). Two-tailed Student’s *t*-test (**c**); ****P < 0.0001. **d** Diagram of the pH dependence test of miRNA uptake in PGECs. **e** Flow cytometry and quantification of apoptotic PGECs after 30 min of incubation at pH 7.4 or 3.5 (*Sidt1*^+/+^, *n* = 4; *Sidt1*^−/−^, *n* = 3). Two-tailed Student’s *t*-test. ns, not significant. **f** Uptake of miRNAs by *Sidt1*^+/+^ or *Sidt1*^−/−^ PGECs after 30-min incubation with miRNAs at the indicated pH, quantified by RT-qPCR (*n* = 9). Two-way ANOVA with Sidak’s post hoc test. ns not significant, ****P < 0.0001. **g** Diagram of the collection of exosomes secreted by PGECs. **h** The miRNA levels in exosomes secreted by *Sidt1*^+/+^ or *Sidt1*^−/−^ PGECs after incubation with miRNAs at pH 7.4 or 3.5 (*n* = 9). Two-way ANOVA with Sidak’s post hoc test. ns, not significant, ****P < 0.0001. **i** Diagram of biological activity measurement of exosomal miRNAs secreted by PGECs. **j** Biological activities of exosome**-**delivered miRNAs in recipient cells measured by a luciferase assay (*n* = 9). Two-tailed Student’s *t-*test. ****P < 0.0001.

To further assess the impact of SIDT1 deficiency on exogenous miRNA uptake, we isolated and cultured PGECs from *Sidt1*^+/+^ and *Sidt1*^−/−^ mice. After incubation with Cy3-labeled miR168a, fluorescence was detected in the cytoplasm of *Sidt1*^+/+^ cells but not in *Sidt1*^−/−^ cells (Fig. 5a); and miR168a was observed only in *Sidt1*^+/+^ cells by in situ hybridization (Fig. 5b). Consistent with this finding, the dramatically decreased uptake of exogenous miRNAs in the *Sidt1*^−/−^ cells was confirmed by RT-qPCR analysis (Fig. 5c).

Furthermore, to mimick the acidic environment in the stomach, the PGECs were cultured under various acidic conditions (Fig. 5d). Notably, incubation with the low-pH medium for the 30-min experimental period did not cause cell death in PGECs (Fig. 5e; Supplementary information, Fig. S4a–d). Interestingly, as shown in Fig. 5f, incubating PGECs with miR156a, miR168a or miR2911 in the medium at pH 4.5, pH 4.0, or pH 3.5 for 30 min dramatically enhanced miRNA uptake in a pH-dependent manner. At pH 3.5, the miR156a, miR168 and miR2911 levels in *Sidt1*^+/+^ cells were increased by approximately 1500-, 70-, and 30-fold, respectively, compared to those under neutral conditions. In contrast, the *Sidt1*^−/−^ cells showed almost no increase in miRNA uptake under the same acidic conditions. We also tested the double-stranded miRNA mimics on the PGECs at pH 7.4 and pH 3.5. At pH 3.5, the double-stranded miR156a and miR168a levels in *Sidt1*^+/+^ cells were increased by ~3-fold, and the double-stranded miR2911 level was not increased. However, the double-stranded miRNA levels in *Sidt1*^−/−^ cells were not significantly changed under the acidic conditions (Supplementary information, Fig. S5). These results indicate that the acidic environment in the stomach can enhance the SIDT1-mediated absorption of food-derived miRNAs.

Previous studies proposed that absorbed exogenous miRNAs can be packaged into exosomes and then carried into cells as functional secreted miRNAs.^1,2,18^ Therefore, to assess whether exogenous miRNAs absorbed by SIDT1 are secreted by PGECs, exosomes were harvested from the culture medium of PGECs after incubation with miRNA at pH 7.4 or 3.5 (Fig. 5g). As shown in Fig. 5h, incubating PGECs with miRNAs in the low-pH (pH 3.5) medium dramatically increased the miRNA levels in exosomes derived from *Sidt1*^+/+^ donor PGECs but failed to do so in *Sidt1*^−/−^ donor PGECs. To functionally assess the effect of secreted miRNAs, we expressed a luciferase reporter in recipient HEK293T cells and then incubated cells with the harvested exosomes (Fig. 5i). The miRNAs in exosomes derived from *Sidt1*^+/+^ donor PGECs significantly inhibited luciferase reporter activity in recipient cells, whereas exosomal miRNAs from *Sidt1*^−/−^ donor PGECs had no inhibitory effect (Fig. 5j). These results suggest that following the enhanced SIDT1-mediated uptake at low pH (pH 3.5), exogenous miRNAs are packaged into exosomes and subsequently secreted as functional miRNAs.

### The antifibrotic effect of orally administered miR2911 is abolished in *Sidt1*-knockout mice

**Fig. 6:**
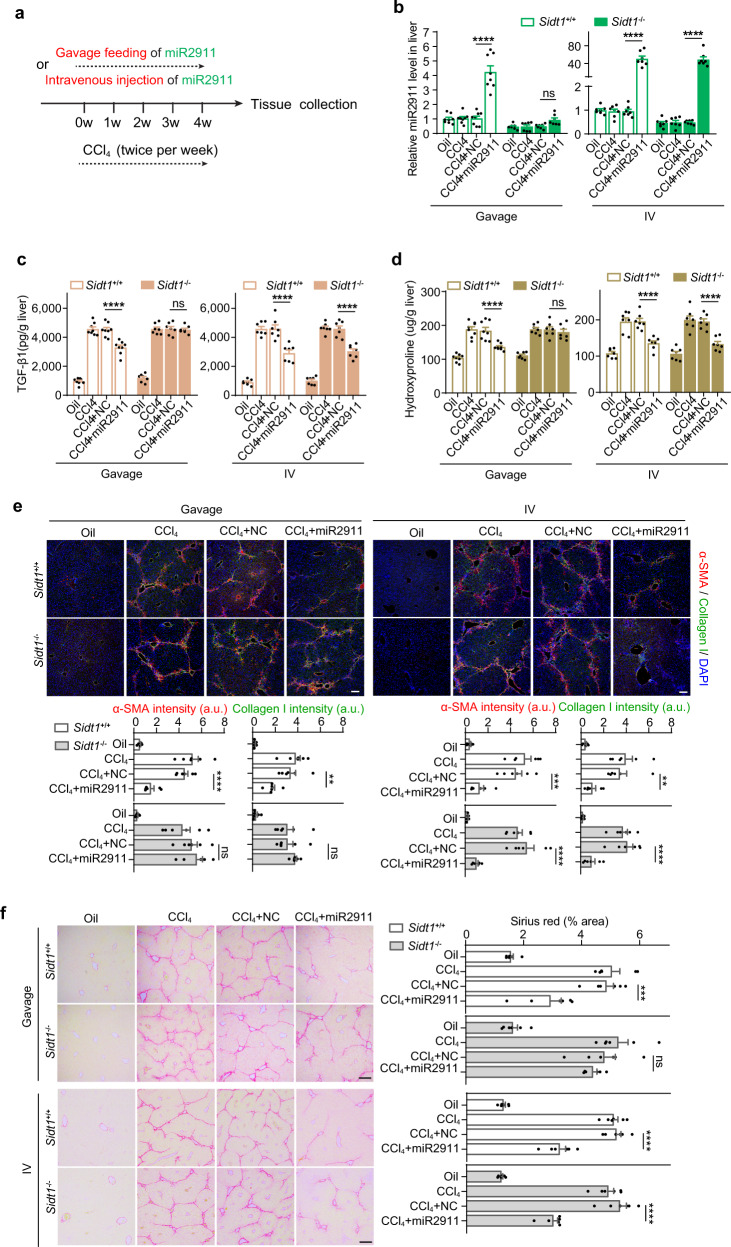
The antifibrotic effect of orally administered miR2911 is abolished in *Sidt1*-knockout mice. **a** Diagram of miR2911 gavage feeding or intravenous injection treatment in a CCl_4_-induced liver fibrosis mouse model. **b**–**d** The miR2911 (**b**), TGF-β1 (**c**) and hydroxyproline (**d**) levels in livers of *Sidt1*^+/+^ or *Sidt1*^−/−^ mice after gavage feeding (*Sidt1*^+/+^, *n* = 8; *Sidt1*^−/−^, *n* = 7) or intravenous injection (*Sidt1*^+/+^, *n* = 7; *Sidt1*^−/−^, *n* = 7) of miR2911. Two-way ANOVA with Sidak’s post hoc test. ns, not significant, ****P < 0.0001. **e** Representative images (upper panel; scale bar, 100 μm) and quantification (bottom panel; *n* = 6) of immunostaining for α-SMA and collagen I in *Sidt1*^+/+^ or *Sidt1*^−/−^ liver tissue sections after gavage feeding or intravenous injection of miRNA. a.u., arbitrary unit. One-way ANOVA with Tukey’s post hoc test. ns, not significant, **P < 0.01, ***P < 0.001, ****P < 0.0001. **f** Representative images (left panel; scale bar, 200 μm) and quantification (right panel; *n* = 6) of Sirius red staining in *Sidt1*^+/+^ or *Sidt1*^−/−^ liver tissue sections after gavage feeding or intravenous injection of miRNA. One-way ANOVA with Tukey’s post hoc test. ns, not significant, ***P < 0.001, ****P < 0.0001.

On the basis of the above in vitro studies, we hypothesized that the dietary miRNAs absorbed via SIDT1 can be secreted and play biological roles in vivo. To test this hypothesis, we first identified mouse and human TGF-β1 as a potential mammalian target of miR2911 (Supplementary information, Fig. S6a–c). We then adopted a CCl_4_-induced liver fibrosis model in which downregulation of TGF-β1 can ameliorate liver fibrosis.^19–21^ As shown in Fig. 6a, mice were intraperitoneally injected with CCl_4_ twice per week for four weeks in combination with daily oral administration of miR2911; in comparison, intravenous (IV) injection of miR2911 was performed as a positive control to “bypass” dietary miRNA absorption via the GI tract. In the gavage groups, dramatically increased miR2911 levels were detected in the livers of *Sidt1*^+/+^ mice (Fig. 6b), and consequently, CCl_4_-induced expression of TGF-β1 was significantly suppressed (Fig. 6c). However, in the livers of *Sidt1*^−/−^ mice, the increase in miR2911 and suppression of TGF-β1 expression were abolished (Fig. 6b, c). Consequently, daily miR2911 administration also significantly decreased the hepatic hydroxyproline level (Fig. 6d), α-smooth muscle actin (α-SMA) level and collagen I level in *Sidt1*^+/+^ mice (Fig. 6e). This finding together with the Sirius red staining results (Fig. 6f) showed that daily miR2911 feeding dramatically inhibited liver fibrosis in *Sidt1*^+/+^ mice. In contrast, oral administration of miR2911 failed to alleviate liver fibrosis in *Sidt1*^−/−^ mice (Fig. 6e, f). In the IV injection groups, elevated miR2911 levels, suppressed expression of TGF-β1, decreased hepatic hydroxyproline levels and alleviated liver fibrosis were all observed in the livers of both *Sidt1*^+/+^ and *Sidt1*^−/−^ mice (Fig. 6b–f). The above results demonstrated that the lack of SIDT1 specifically impairs the absorption and pharmaceutical effects of orally administered miRNAs.

## Discussion

For decades, the primary function of the stomach has been thought to aid in food breakdown with mechanical churning and secretion of hydrochloric acid and pepsin.^8^ Due to the lack of the typical villus-type absorptive membrane and nutrient transport, the stomach is considered to be a poor absorptive area in the GI tract. Only water and a few highly lipid-soluble substances, such as alcohol and some drugs (e.g., aspirin), can be absorbed in small quantities in the stomach by passive diffusion.^8^ Most nutrients, including digested monosaccharides, amino acids, single nucleotides and mineral salts, are mainly absorbed in the small intestine, which contains abundant digestive enzymes secreted by digestive glands such as the pancreas.^8^ In this study, we clearly show that dietary miRNA absorption occurs in the stomach and that stomach-enriched SIDT1 is the key transporter. This newly discovered physiological role of the stomach expands the understanding of the mammalian digestive system.

Since naked RNAs are highly susceptible to ribonuclease (RNase)-mediated degradation in in vivo environments, one important consideration for in vivo delivery of therapeutic RNAs is the stability of RNAs, which represents a critical limitation in the therapeutic application of RNA molecules.^6^ Although accumulated evidence shows the existence of intact dietary miRNAs in mammalian hosts,^22,23^ the absorption of dietary miRNAs in the animal GI tract has been frequently questioned, mainly due to the assumed poor stability of naked miRNAs in the GI tract, where many digestive RNases exist. The hostile environment of the gut was thought to pose significant barriers to the stability of orally administered plant miRNAs.^24^ However, the highly acidic internal environment of the stomach, in which RNases are barely active, provides an optimal location for absorption of stable dietary miRNAs. The dietary miRNAs first enter the stomach and are absorbed there, avoiding degradation in the small intestine. Our study identified the stomach as the primary site of small RNA absorption.

SIDT1 was initially proposed as a dsRNA transporter in mammalian cells.^16^ Consistent with this notion, we found that uptake of single-stranded mature miRNA was significantly lower than that of double-stranded miRNA mimics at pH 7.4 in cultured cells. Notably, in our in vivo study, to achieve a similar level of exogenous plant miRNA in serum, the dose of gavage-fed synthetic miRNA was much higher than that of natural plant miRNA in rice or honeysuckle decoction. Although double-stranded mature miRNAs do not naturally exist in the diet,^25,26^ complementary RNA fragments of plant miRNAs exist in plants. Further study is required to explore whether these fragments could bind miRNAs to form a double-stranded or partially double-stranded structure, which would be a more suitable substrate for SIDT1, thus leading to a higher absorption efficiency of miRNAs derived from natural food than that of pure synthetic miRNAs. In our in vitro study, the absorption efficiency of miR156a, 168a and 2911 is largely different, suggesting that SIDT1 may mediate the uptake of exogenous miRNAs selectively. The detailed biochemical and biophysical properties of SIDT1 involved in substrate preference and sequence selectivity require further studies at the single-molecule or single-channel level. Besides, it is worth noting that the uptake of mature single-stranded plant miRNA by SIDT1 was greatly enhanced under acidic culture conditions that mimicked the acidic stomach environment, while the effect on double-stranded miRNA mimics was not that obvious. It would be interesting to further study whether acidic conditions could induce conformational or structural changes in mature miRNA, e.g., facilitating the formation of a double-stranded structure. On the other hand, we cannot rule out the possibility that acidic culture conditions in vitro might cause a conformational or structural change in SIDT1 toward the natural protein structure under physiological conditions, leading to an enhanced binding capacity for substrates or altered channel permeability.^27–29^ In addition, a small portion of miRNA absorption capacity remains upon SIDT1 knockout both in vivo and in vitro, suggesting that SIDT1-independent mechanisms may also contribute to exogenous miRNA absorption. For example, it has been shown that exosomes from dietary sources such as bovine milk can mediate the miRNA absorption by non-bovine species^30^ and heat-stable decoctosomes (exosome-like nanoparticles) from decoctions of herbal medicines can mediate the small RNA absorption via oral administration.^31^ Nevertheless, the current study suggests that SIDT1-dependent uptake might be the major mechanism of dietary miRNA absorption. It is interesting to study alternative mechanisms in future.

**Fig. 7:**
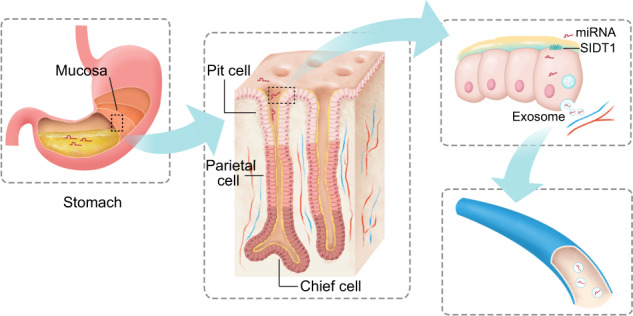
A graphical model of the absorption and secretion of dietary miRNAs in the stomach. During feeding, the dietary miRNAs are released into the lumen of the stomach by mechanical digestion. In such an acidic environment, mature miRNAs escape from degradation by nuclease and the miRNAs are efficiently absorbed via SIDT1 protein which locates at the plasma membrane of pit cells. The intracellular dietary miRNAs are transported into the multivesicular bodies and the dietary miRNA-containing exosomes are secreted from pit cells. Then the secreted exosomes are pooled into the circulatory system and delivered to other tissues and organs.

In the past few decades, compared with conventional drugs such as small molecules and proteins, RNA-based therapies have developed rapidly and exhibit numerous advantages, such as easier and faster design and production, cost effectiveness, expanded range of therapeutic targets, and flexible combinations of drug cocktails for personalized treatment.^6,32,33^ RNA-based therapeutic agents, including siRNA, miRNA, and mRNA, can knock down gene expression, regulate target genes, or induce the expression of specific genes.^25,34^ However, since it is extremely difficult for a naked RNA that is too large and highly charged to pass through the cell membrane by free diffusion,^6^ efforts have been continuously made to develop safe and efficient RNA delivery systems, such as carriers or vectors. Currently, the most commonly used vectors for miRNA delivery include viral and nonviral vectors. However, viral vectors, such as adeno-associated viruses and lentiviruses,^35^ and nonviral vectors, such as lipid- or polymer-based nanoparticles and conjugate platforms,^36,37^ have several disadvantages, such as immunogenicity,^38^ cytotoxicity,^39^ insertional mutagenesis^35^ and other side effects,^40^ and limitations, including limited delivery efficiency,^41,42^ high cost,^39^ and difficulties of vector production.^42,43^ In this study, we found that exogenous dietary miRNAs can be transported into pit cells by the intrinsic carrier protein SIDT1 in the stomach and then secreted as functional entities in exosomes, natural nanoparticles that can protect these miRNAs from degradation in the bloodstream and aid their cellular uptake (Fig. 7). This natural mammalian absorption pathway of dietary miRNAs can be easily harnessed for oral delivery of therapeutic miRNAs, which can be a significant direction in the future development of RNA-based medicine.

In summary, our findings not only reveal the mechanism of dietary miRNA absorption and demonstrate a novel physiological function of the mammalian stomach, but also shed light on developing oral delivery-based small RNA therapeutics.

## Materials and methods

### Animals and reagents

All experimental mice were maintained on a C57BL/6 J background. Mice were group-housed at 22–24 °C with a 12-h light–dark cycle, and with ad libitum access to a regular chow diet and water in a standard animal facility at Nanjing University (Nanjing, China). All animals were handled in accordance with the NIH Guidelines for the Care and Use of Laboratory Animals and all mouse protocols were approved by the Institutional Animal Care and Use Committee of Nanjing University. *Sidt1*^−/−^ mice (strain *Sidt1*^tm1(KOMP)Vlcg^) were obtained from the Knockout Mouse Project (KOMP) Repository at University of California, Davis (CA, USA). The primers for mouse genotyping were as follows: *Sidt1*^+/+^ (forward: 5′-gaagcagaggtgctcagaaatgg-3′, reverse: 5′-gcttgtctcgccacaactcttgtc-3′), *Sidt1*^−/−^ (forward: 5′- gcagcctctgttccacatacacttca-3′, reverse: 5′-ttgcatgtcattcgttaccttgacc-3′). All synthetic miRNAs were customized from TaKaRa (Dalian, China), and the fluorescently labeled miRNAs were customized from RiboBio (Guangzhou, China). The miRNA sequences were: miR156a (5′-ugacagaagagagugagcac-3′), miR168a (5′-ucgcuuggugcagaucgggac-3′), miR2911 (5′-ggccgggggacgggcuggga-3′), lgi-miR-133-5p (5′-agcugguugaaauugggccaaau-3′). All the reagents used in this study were of analytical grade, HPLC grade or molecular biology grade.

### Isolation of PGECs

PGECs were isolated from both male and female mice (P7) by a method described previously^44,45^ with some modifications. Briefly, *Sidt1*^+/+^ or *Sidt1*^−/−^ mouse pups (P7) were sacrificed after fasting for 12 h, the gastric epithelial tissues were separated from the stomach, divided into 1–2 mm pieces and digested with 0.125% trypsin-EDTA (Gibco) for 5 h at 4 °C and 20–25 min at 37 °C continuously, followed by termination with fetal bovine serum (FBS; Gibco). The digested tissue pieces were filtered through a 70-μm-nylon mesh (Thermo Fisher Scientific), and then centrifuged at 400× *g* for 3 min. The harvested cells were plated on PDL-coated cell culture dishes with M199 medium (Hyclone, SH30253.02) supplied with 10% FBS, 1% penicillin-streptomycin (Gibco), 100 mg/L heparin (MCE, HY-17567), 1× ECGS (ScienCell^TM^, Cat# 1052) and 10 μg/L EGF (Novoprotein, C026). After 2 days of culture, the primary cells were used in the following experiments.

### Isolation of mouse peritoneal macrophages

Mouse peritoneal macrophages were isolated and cultured as described previously.^46,47^ Briefly, mice were sacrificed and disinfected with 75% ethanol. A small incision was made and the skin was pulled firmly to expose the peritoneal wall. Then, 5–8 mL of PBS was injected into peritoneal cavity, followed by abdominal massage for 30 s. The peritoneal fluid was then withdrawn and centrifuged at 300× *g* for 3 min. The cell pellet was washed with PBS and centrifuged at 300× *g* for 3 min. The cell pellet was resuspended in RPMI medium supplied with 10% FBS and 1% penicillin-streptomycin, and cultured at 37 °C with 5% CO_2_.

### Cell lines and culture conditions

HEK293T cells and HepG2 cells (ordered from Type Culture Collection of Chinese Academy of Sciences) were cultured in high-glucose DMEM (Thermo Fisher Scientific, Cat# 11965118) supplemented with 10% FBS and 1% penicillin-streptomycin at 37 °C with 5% CO_2_ and routinely passaged 2–3 times a week. Both cell lines were authenticated by STR and confirmed to be mycoplasma free.

### Illumina deep sequencing after oxidation of small RNAs with periodate

Briefly, small RNAs were extracted from the mouse liver using miRNeasy Mini kit (QIAGEN, Cat# 217004) according to the manufacturer’s instructions. A 100-μL mixture consisting of 20 μg of small RNA and 10 mM NaIO_4_ was incubated at 0 °C for 40 min in the dark. The oxidized RNA was precipitated with ethanol, rinsed once with 75% ethanol, air-dried, and dissolved in 50 μL of DEPC water. An equal volume of 2 M Lys-HCl was added, and the solution was incubated at 45 °C for 90 min for β-elimination. The solution was then precipitated twice with ethanol, rinsed once with 75% ethanol, air-dried, and dissolved in DEPC water. The samples from three different individuals with the same genotype were mixed and subjected to deep sequencing. Illumina deep sequencing of RNA samples was performed by BGI (Shenzhen, China). After removing low-quality and contaminated sequences and trimming the adaptor sequences of raw data, the clean reads with a length of 18–30 nucleotides remained for further analysis. These reads were then compared to known miRNA precursors and mature miRNAs in miRBase 16.0 database (www.mirbase.org) to identify conserved plant miRNAs based on the Smith-Waterman algorithm (2 shifts and 2 mistakes allowed). All data have been uploaded to the GEO database (GEO accession number: GSE132152).

### RNA isolation and reverse transcription PCR

Total RNA was extracted from tissues and cultured cells using TRIzol reagent (Thermo Fisher Scientific, Cat# 15596026) according to the manufacturer’s instructions. Total RNA from serum was extracted using phenol-chloroform method (Sigma-Aldrich, P4682).

For semi-quantitative analysis of mRNA, 1 μg of total RNA was reverse transcribed to cDNA with PrimeScript™ RT reagent Kit (TaKaRa, RR047A). The sequences of the PCR primers were as follows: mmu-*Sidt1*-mRNA (forward: 5′-tcctcccgctaccagtct-3′, reverse: 5′-cacatccaggtcatcatcc-3′), mmu-*Gapdh*-mRNA (forward: 5′-tgaagcaggcatctgaggg-3′, reverse: 5′-cgaaggtggaagagtgggag-3′). PCR products were detected by agarose gel electrophoresis.

For quantification of mature miRNAs, RT-qPCR was performed using TaqMan^®^ miRNA probes (Thermo Fisher Scientific) according to the manufacturer’s instructions. qPCR was performed on a LightCycler 480 Real-time PCR System (Roche Diagnostics). U6 snRNA was used for normalization of miRNA expression in tissues, cells and exosomes; the relative level of each miRNA was determined using the 2^−ΔΔCt^ method.^48^ For calculation of the miRNA level in serum, a series of synthetic miRNA oligonucleotides at known concentrations were reverse transcribed and amplified. The level of each miRNA was then calculated according to the standard curve.

### Gavage feeding

For analysis of the absorption of synthetic dietary miRNAs in *Sidt1*^+/+^ and *Sidt1*^−/−^ mice, adult male mice were gavage fed synthetic miR156a, miR168a or miR2911 (40 nmol/kg body weight). Serum and tissue samples were collected, and total RNA was extracted. The control group (named 0 h) was euthanized after 24 h of fasting with gavage feeding of saline.

### Feeding mice with natural foods

To mimic a physiological dosage, adult male mice were gavage fed with honeysuckle decoction (20 mL/kg body weight). Before feeding, serum samples were collected via the tail vein (0 h). At 6 h or 12 h after gavage feeding, serum samples were collected, and total RNA was extracted. In a separate experiment, adult male mice were fed rice or chow; serum samples were collected via the tail vein just before rice feeding (0 h). Serum samples were collected at 3 h, 6 h or 12 h, and total RNA was extracted. For analysis of the function of miR168a in *Sidt1*^+/+^ and *Sidt1*^−/−^ mice, adult male mice were fed rice for 3 days; serum and liver samples were collected for analysis of LDL cholesterol and LDLRAP1 protein, respectively.

### Western blot

Samples of tissues and cultured cells were lysed in Cell Extraction Buffer (Thermo Fisher Scientific, FNN0011) and centrifuged at 12,000× *g* for 10 min at 4 °C. The supernatant fraction was collected, and the protein concentration was quantified by Pierce™ BCA Protein Assay Kit (Thermo Fisher Scientific, Cat# 23225). Proteins were separated on SDS-polyacrylamide gels and transferred to polyvinylidene difluoride membranes. The membranes were blocked for 1 h, followed by overnight incubation with primary antibodies at 4 °C. After the membranes were washed, they were incubated with the appropriate secondary antibody conjugated to horseradish peroxidase (Cell Signaling Technology) at room temperature for 1 h and then the protein was detected with an enhanced chemiluminescence reagent (Thermo Fisher Scientific). The following primary antibodies were used: anti-SIDT1 (1:500, Abcam, ab134992), anti-LDLRAP1 (1:1000, Lifespan Biosciences, LS-C20125), and anti-GAPDH (1:2000, Cell Signaling Technology, Cat# 2118).

### Analysis of serum LDL cholesterol

Mouse serum samples were assayed using commercially available kits (Daiichi Pure Chemicals) and a clinical chemistry analyzer (HITACHI 7600, Hitachi Koki).

### Immunofluorescence staining and plasma membrane labeling

Sections of different tissues were fixed in 4% paraformaldehyde (PFA), washed with PBS for 30 min and blocked for 1 h at room temperature. The samples were then incubated with primary antibody at 4 °C overnight, followed by incubation with the Alexa Fluor 594-conjugated or 488-conjugated secondary antibody (1:1000, Thermo Fisher Scientific) in a dark room at room temperature. Nuclei were counterstained with DAPI (Beyotime, C1002) for 5 min and then washed in PBS. The following primary antibodies were used: anti-SIDT1 (1:200, Abcam, ab134992), anti-Pepsin C (1:50, Santa Cruz Biotechnology, sc-51188), anti-Atp4b (1:500, Abcam, ab2866), anti-Calpain-8 (1:200, Abcam, ab28215; for double labeling with SIDT1, adjacent stomach sections were stained by anti-Calpain-8 or anti-SIDT1, respectively), anti-α-SMA (1:100, BosterBio, BM0002), and anti-Collagen I (1:100, Abcam, ab34710). Adherent cultured PGECs were washed and stained with the PKH67 green fluorescent cell Linker Kit for General Cell Membrane Labeling (Sigma, PKH67GL) according to the manufacturer’s instructions. When membrane labeling was completed, the staining solution was removed, and cells were washed and fixed in 4% PFA. Plasma membrane-labeled cells were then immunofluorescently stained with antibodies according to the methods mentioned above. All fluorescence images were captured on a Leica TCS SP8 MP confocal microscope (Leica Microsystems).

### Pylorus ligation experiments

Mice were deprived of food for 24 h (with free access to water) and then anesthetized with isoflurane, and the abdomen was opened. The pylorus was then ligated by applying silk suture material firmly around the junction between the pylorus and the duodenum, followed by abdominal closure as described previously.^49^ In the sham pylorus ligation (control) group, the pylorus was exposed and left untreated, followed by abdominal closure. Mice were then gavage fed with synthetic miRNAs after revival, and the serum samples were collected after 3 h. For the validation of the pylorus ligation surgery, mice were gavage fed with a 33.3% glucose solution (1 g/kg body weight). Blood glucose levels of the sham and pylorus-ligated mice were assessed at different time points after glucose gavage.

For analysis of the kinetic process of plant miRNA absorption through the stomach, mice were gavage fed with Cy5-labeled synthetic miR168a at a dose of 100 nmol/kg body weight. Mice were euthanized at 3 h, 6 h after gavage feeding. The stomach was extensively washed with saline solution and immediately imaged ex vivo using the Maestro EX in vivo fluorescence imaging system (Cambridge Research & Instrumentation, CRi) with the software platform Maestro EX 3.0. The signals were collected in the 675–750 nm channel with excitation at 635 nm.

For analysis of the plant miRNA distribution in stomach, lung and liver after gavage feeding with Cy3-labeled synthetic miR168a at a dose of 100 nmol/kg body weight, mice were euthanized at 3 h after gavage feeding. The tissues were carefully washed, fixed in PFA, sectioned and observed under a Leica TCS SP8 MP confocal microscope.

### In situ hybridization

The ViewRNA^®^ miRNA ISH Cell Assay Kit and miR168a probe were purchased from Thermo Fisher Scientific (Cat# QV-CM0001). According to the manufacturer’s guidance, the PGECs (*Sidt1*^+/+^ and *Sidt1*^−/−^) were plated onto the coverslips in 24-well plates. After incubation with miR168a (40 pmol/mL) for 30 min, the cells were extensively washed with PBS three times. The cells were fixed with 4% fresh formaldehyde, followed by fresh EDC crosslinking. Next, the cells were permeabilized with detergent solution provided by kits. After digestion with protease for 10 min, the cells were hybridized with miR168a probe for 3 h. After the samples were washed with Wash Buffer 3 times, storage buffer was added to the wells, and the cells were stored at 4 °C overnight. On the next day, the cells were sequentially hybridized with PreAmplifier and Amplifier. After extensive washing, the miR168a signal was developed with Fast Red substrate followed by DAPI staining. The mounted coverslips were imaged on a Leica TCS SP8 MP confocal microscope.

For *Sidt1* mRNA in situ hybridization on tissue samples, freshly prepared GI tract tissue slides were used. The mouse *Sidt1* mRNA RNAscope^®^ probe (Cat# 425471) and the in situ hybridization kit were purchased from the Advanced Cell Diagnostics Company. The hybridization was performed according to the manufacturer’s instructions. All the steps were the same as described in the protocol. The samples were imaged on a confocal microscope (Fluoview FV1000, Olympus).

### miRNA uptake assay in cultured cells

The PGECs were exposed to miRNA (40 pmol/mL) in medium containing 2% FBS for different periods of time. Prior to harvest, cells were extensively washed and incubated with FBS-free medium containing 0.2 mg/mL RNase A (Thermo Fisher Scientific, Cat# 12091021) for 1 h to digest the extracellularly attached miRNAs. Cells were washed three times, and total RNA was extracted. For fluorescent signal detection, cells were incubated with Cy3-labeled miR168a for 30 min, washed extensively, and then observed under a Leica TCS SP8 MP confocal microscope.

### Low pH treatment

The pH of the culture medium was adjusted with hydrochloric acid to 3.5, 4.0 or 4.5, and an unmodified culture medium (approximately pH 7.4) was used as a control. Synthetic miRNAs were directly added to the buffered medium at different pH values (40 pmol/mL), and the resulting mixture was then added to cultured cells for 30 min. After incubation with miRNA in medium at different pH, the cells were washed three times and incubated with FBS-free medium containing RNase A at 37 °C for 1 h to remove extracellularly attached miRNAs.

### Cell apoptosis assay

Cell apoptosis was analyzed by flow cytometry with a FITC-Annexin V Apoptosis Detection Kit (Yeasen Biotech). Before plating cells to the dishes, the dead PGECs were first removed by the Dead Cell Removal Kit (Miltenyi Biotec, Cat# 130-090-101). After the low pH treatment, the cells were harvested by mild trypsin digestion. The FITC-Annexin V and propidium iodide were used for double staining in accordance with the manufacturer’s instructions, followed by Gallios flow cytometry (Beckman Coulter). Kaluza for Gallios acquisition software (Beckman Coulter) was used to acquire the data and at least 5000 events were collected in each analysis. We distinguished living cells, dead cells, early apoptotic cells, and apoptotic cells. The relative proportion of early apoptotic cells and apoptotic cells was combined as the target of our comparison.

### Cell viability assay

The PGECs were cultured and then incubated with medium at different pH values for 30 min. After the treatment, cell viability was analyzed by the Cell Counting Kit-8 (Dojindo, CK04) according to the manufacturer’s instructions. The absorbance at 450 nm was read on a SpectraMax M2e (Molecular Devices).

### Lactate dehydrogenase (LDH) cytotoxicity assay

The PGECs were cultured and then incubated with medium at different pH values for 30 min. After the treatment, the old medium was replaced with fresh medium at normal pH, and then, the cell culture medium was collected after 1 h for LDH determination with LDH Cytotoxicity Assay Kit (Beyotime, C0016) according to the manufacturer’s instructions. Briefly, the medium was transferred to a new plate and mixed with Reaction Mixture. After a 30-min incubation at room temperature, reactions were stopped by adding Stop Solution. Absorbance at 490 nm was measured on a SpectraMax M2e (Molecular Devices) to determine LDH activity. LDH release reflected cell death.

### Live/dead cell staining

The LIVE/DEAD^TM^ Imaging Kit (Thermo Fisher Scientific, R37601) was used to detect the live and dead cells. Briefly, cells were cultured on coverslips and then incubated with medium at different pH values for 30 min. The cells were washed with PBS and dyed according to the manufacturer’s instructions. The labeled cells were photographed under an inverted microscope (IX71, Olympus). The live cells fluoresce green, and dead cells fluoresce red.

### Exosome collection

The cells were incubated with miRNA (400 pmol/mL) in a medium at pH 7.4 or pH 3.5 for 30 min. After incubation with miRNA, the cells were extensively washed and incubated with RNase A for 1 h to digest the extracellularly attached miRNAs. Then, the cells were washed and cultured with fresh medium containing 10% FBS. After 36-h culturing, exosomes were isolated from cell culture medium using an Exosome Isolation Kit (RiboBio, C10130) according to the manufacturer’s instructions. Briefly, after cell debris and shedding vesicles were removed by centrifugation at 2000× *g* for 30 min and then at 10,000× *g* for 1 h, the supernatant was mixed with isolation reagent and incubated at 4 °C overnight. The solution was centrifuged at 3000× *g* for 1 h (all steps were performed at 4 °C). Exosome pellets were collected and resuspended in PBS. The concentration of exosomes was determined by measuring the protein concentration using a BCA assay kit. The total RNA was extracted for measurement of miRNA concentration. To test the function of miRNAs delivered by exosomes to recipient cells by the luciferase assay, the gastric cell-secreted exosomes were added to the HEK293T cells and incubated for 8 h before luciferase plasmid transfection.

### Plasmid construction and luciferase assay

For luciferase reporter assays of exosomes, synthetic DNA fragments corresponding to the complementary sequences of miR156a, miR168a or miR2911 were ligated to the 3′-UTR of the pMIR-REPORT plasmid (Thermo Fisher Scientific). The HEK293T cells were incubated with exosomes resuspended in medium containing 2% FBS for 8 h, washed extensively with PBS, and then transfected with 0.25 μg of firefly luciferase reporter plasmid and 0.25 μg of β-galactosidase (β-gal) control plasmid (Thermo Fisher Scientific) per well in 24-well plates. At 10 h post transfection, cells were analyzed using a luciferase assay kit (Promega). For validation of miR2911 targeting TGF-β1, a 729-bp segment of the 3′-UTR of human TGF-β1 that contained the presumed miR2911 binding site was generated and inserted into the pMIR-REPORT plasmid. To test the binding specificity, another 529-bp segment without the binding site was generated as the mutant control. Briefly, 0.25 μg of firefly luciferase reporter plasmid, 0.25 μg of β-gal control plasmid, and 50 pmol of miR2911 were transfected into cells per well in 24-well plates for 6 h. At 24 h post transfection, the cells were assayed using a luciferase assay kit (Promega).

### Enzyme linked immunosorbent assay (ELISA)

To validate whether miR2911 directly targets TGF-β1, the HepG2 cells and mouse peritoneal macrophages were plated into 6-well plates, and exposed to NC miRNA or miR2911 (2 nmol/mL) in medium containing FBS. After 24 h, cell culture supernatant was collected and TGF-β1was measured using TGF-β1 Human ELISA kit (Invitrogen, BMS249-4) or TGF-β1 Mouse ELISA kit (Invitrogen, BMS608-4), respectively, according to the manufacturer’s instructions.

### CCl_4_-induced liver fibrosis model

For analysis of the function of synthetic dietary miR2911 in *Sidt1*^+/+^ and *Sidt1*^−/−^ mice, a CCl_4_-induced liver fibrosis model was established. Male *Sidt1*^+/+^ and *Sidt1*^−/−^ mice were intraperitoneally injected with 25% CCl_4_ solution in sterile olive oil at a dose of 1 mL CCl_4_/kg body weight twice per week for four weeks. One day after the last injection of CCl_4_, mice were sacrificed. Fresh liver tissues were collected to detect the levels of miR2911, TGF-β1 (4 A Biotech, CME0020) and hydroxyproline (Nanjing Jiancheng Bioengineering Institute, A030-2). Paraffin sections were prepared from the remaining livers. Liver fibrosis was further evaluated by Sirius red staining and α-SMA/collagen I immunofluorescence staining. For analysis of the antifibrotic effect of oral miR2911, *Sidt1*^+/+^ and *Sidt1*^−/−^ mice were randomly divided into four groups. Group 1 mice were treated with olive oil only for 4 weeks; group 2 mice were treated with CCl_4_ for 4 weeks; group 3 mice were treated with CCl_4_ for 4 weeks in combination with gavage feeding of NC miRNA (40 nmol/kg body weight) daily; and group 4 mice were treated with CCl_4_ for 4 weeks in combination with gavage feeding of miR2911 (40 nmol/kg body weight) daily. As an experimental control, an IV injection of miR2911 was performed independently. Group 1 and group 2 mice were treated with olive oil or CCl_4,_ respectively; group 3 and group 4 mice were treated with CCl_4_ for 4 weeks in combination with injection of NC miRNA or miR2911 (280 nmol/kg body weight) into the tail vein once a week, respectively.

### Statistics

Data are presented as means ± SEM. Statistical analyses were performed using GraphPad PRISM 8 software (GraphPad). Imaging data analyses were performed with ImageJ (NIH). *P* values were calculated using two-tailed Student’s *t*-test to compare two groups, one-way ANOVA for experiments consisting of multiple groups, and two-way ANOVA for experiments involving two independent variables. For all experiments, all stated replicates are biological replicates. For in vivo studies, mice were randomly assigned to treatment groups.

## Supplementary information

Supplementary Figure S1

Supplementary Figure S2

Supplementary Figure S3

Supplementary Figure S4

Supplementary Figure S5

Supplementary Figure S6
