# Supplementary material for: SIDT1-dependent absorption in the stomach mediates host uptake of dietary and orally administered microRNAs
Source: Cell Res. 2020 Aug 17;31(3):247–58. doi: 10.1038/s41422-020-0389-3 (PMC8026584; doi:10.1038/s41422-020-0389-3)
Supplement: Supplementary file 1 — Supplementary Figure S1 [file 41422_2020_389_MOESM1_ESM.pdf]

Supplementary information, Figure S1

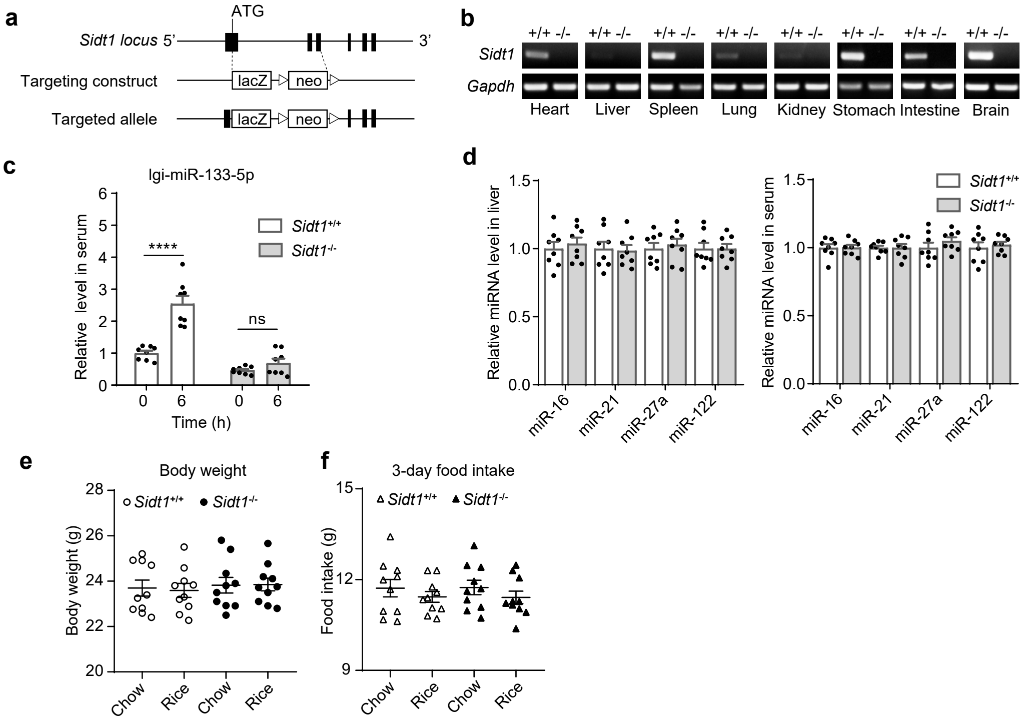

Fig. S1 Validation and characterization of Sidt1 knockout mice. **a,** Diagram of the *Sidt1* gene knock-out. The first three exons of *Sidt1* were replaced with a LacZ gene and a floxed neo gene by homologous recombination. **b,** The knockout efficiency of *Sidt1* gene was validated by RT-PCR. *Sidt1* mRNA was undetectable in *Sidt1*$^{-/-}$ mice tissues. **c,** The lgi-miR-133-5p level in mouse serum following gavage feeding with synthetic lgi-miR-133-5p (n=8 ± SEM). Two-way ANOVA analysis with Sidak's *post hoc* test; ns, not significant, **** P <0.0001. **d,** The levels of endogenous miRNAs detected by RT-qPCR in the liver or serum from *Sidt1*$^{+/+}$ or *Sidt1*$^{-/-}$ mice (n=8 ± SEM). **e,** The body weight of mice fed with chow or rice (n=10 ± SEM). **f,** The food intake of chow or rice (n=10 ± SEM).
