# Supplementary material for: SIDT1-dependent absorption in the stomach mediates host uptake of dietary and orally administered microRNAs
Source: Cell Res. 2020 Aug 17;31(3):247–58. doi: 10.1038/s41422-020-0389-3 (PMC8026584; doi:10.1038/s41422-020-0389-3)
Supplement: Supplementary file 2 — Supplementary Figure S2 [file 41422_2020_389_MOESM2_ESM.pdf]

Supplementary information, Figure S2

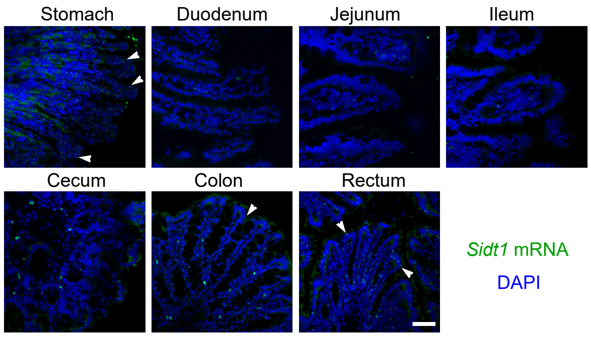

Fig. S2 Characterization of tissue distribution of mouse *Sidt1* mRNA. The *in situ* hybridization signals (green puncta, indicated by arrowhead) of *Sidt1* mRNA in mouse GI tract tissues. Scale bar, 50 μm.
