# Supplementary material for: SIDT1-dependent absorption in the stomach mediates host uptake of dietary and orally administered microRNAs
Source: Cell Res. 2020 Aug 17;31(3):247–58. doi: 10.1038/s41422-020-0389-3 (PMC8026584; doi:10.1038/s41422-020-0389-3)
Supplement: Supplementary file 3 — Supplementary Figure S3 [file 41422_2020_389_MOESM3_ESM.pdf]

Supplementary information, Figure S3

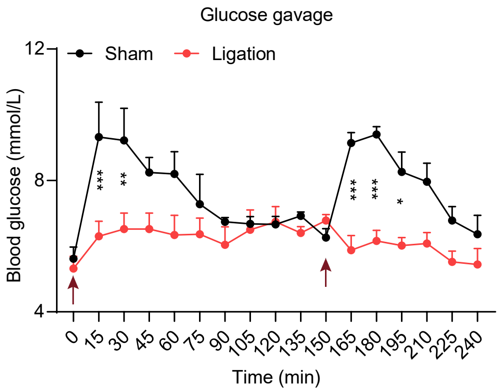

Fig. S3 Blood glucose monitoring to validate the pylorus ligation operation.

The blood glucose levels of the sham and pylorus-ligated mice were monitored at 15 min intervals for 240 min between and after twice glucose gavage (pointed by the arrow) (n=5 ± SEM). Two-way ANOVA analysis with Sidak's *post hoc* test; *P<0.05, **P<0.01, ***P<0.001.
