# Supplementary material for: SIDT1-dependent absorption in the stomach mediates host uptake of dietary and orally administered microRNAs
Source: Cell Res. 2020 Aug 17;31(3):247–58. doi: 10.1038/s41422-020-0389-3 (PMC8026584; doi:10.1038/s41422-020-0389-3)
Supplement: Supplementary file 4 — Supplementary Figure S4 [file 41422_2020_389_MOESM4_ESM.pdf]

Supplementary information, Figure S4

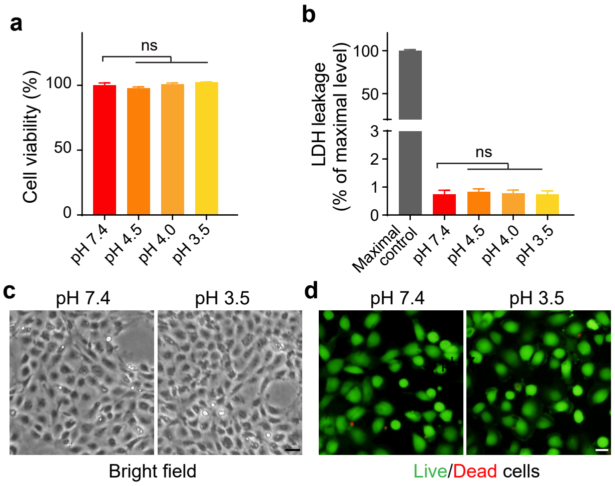

Fig. S4 Analysis of the cytotoxicity of acidic conditions on PGECs. **a,** Cell viability assay, the cell viabilities were examined by CCK-8 assay kit after 30-min exposure to different low pH conditions (n=12 ± SEM). One-way ANOVA analysis with Tukey's *post hoc* test; ns, not significant. **b,** LDH cytotoxicity assay, the LDH released into the culture supernatant by cells was detected 1 h after 30-min exposure to different low pH conditions (n=12 ± SEM), and the values are normalized with the maximal LDH release control group (n=6 ± SEM). One-way ANOVA analysis with Tukey's *post hoc* test; ns, not significant. **c,** Phase-contrast graphs of PGECs after incubation with the medium at pH 7.4 or 3.5 for 30 min. Scale bar, 10 µm. **d,** Live/Dead cell staining, the fluorescence images of PGECs after incubation with the medium at pH 7.4 or 3.5 for 30 min. Scale bar, 10 µm.
