# Supplementary material for: SIDT1-dependent absorption in the stomach mediates host uptake of dietary and orally administered microRNAs
Source: Cell Res. 2020 Aug 17;31(3):247–58. doi: 10.1038/s41422-020-0389-3 (PMC8026584; doi:10.1038/s41422-020-0389-3)
Supplement: Supplementary file 5 — Supplementary Figure S5 [file 41422_2020_389_MOESM5_ESM.pdf]

Supplementary information, Figure S5

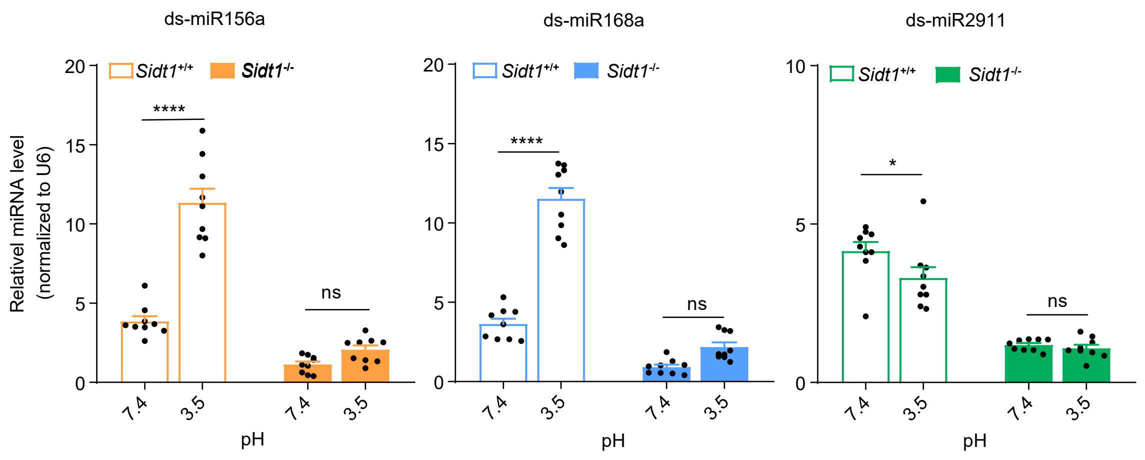

Fig. S5 Double-stranded miRNA mimics are taken up by PGECs through SIDT1 in a pH-dependent manner. Uptake of double-stranded miRNAs (miR156a, miR168a and miR2911) by *Sidt1*$^{+/+}$ or *Sidt1*$^{-/-}$ PGECs after 30-min incubation of miRNA mimics at pH 7.4 and pH 3.5, quantified by RT-qPCR (n=9 ± SEM). Two-way ANOVA with Sidak's *post hoc* test; ns, not significant, * P<0.05, **** *P*<0.0001.
