# Supplementary material for: SIDT1-dependent absorption in the stomach mediates host uptake of dietary and orally administered microRNAs
Source: Cell Res. 2020 Aug 17;31(3):247–58. doi: 10.1038/s41422-020-0389-3 (PMC8026584; doi:10.1038/s41422-020-0389-3)
Supplement: Supplementary file 6 — Supplementary Figure S6 [file 41422_2020_389_MOESM6_ESM.pdf]

Supplementary information, Figure S6

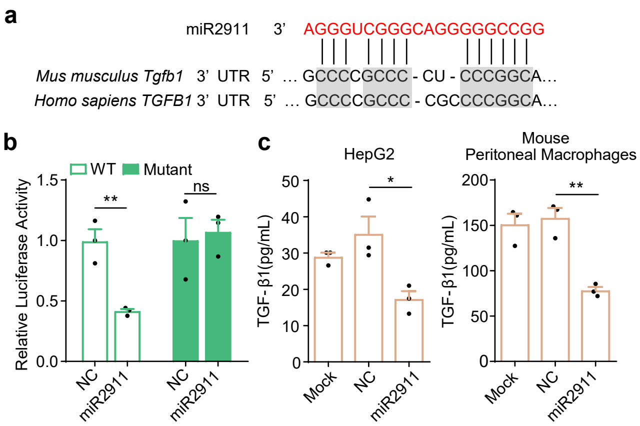

Fig. S6 Identification of TGF-β1 as a target gene of miR2911 *in vitro*. **a,** Schematic description of the base pairing between miR2911 and TGF-β1 mRNA (mouse and human). **b,** Luciferase activity of HEK293T cells co-transfected with firefly luciferase reporters containing either WT or mutant TGF-β1 3'-UTR, and miR2911 or NC miRNA (n=3 ± SEM). Two-tailed Student's *t*-test; ns, not significant, ***P<0.01*. **c,** The reduction of TGF-β1 expression by miR2911 incubation in HepG2 cells (n=3 ± SEM) and mouse peritoneal macrophage cells (n=3 ± SEM). One-way ANOVA analysis with Tukey's *post hoc* test; **P*<0.05, ***P*<0.01.
